# Estimation of optic nerve sheath diameter on an initial brain computed tomography scan can contribute prognostic information in traumatic brain injury patients

**DOI:** 10.1186/cc12589

**Published:** 2013-03-27

**Authors:** Aurélien Legrand, Patrick Jeanjean, Fanny Delanghe, Johann Peltier, Benoit Lecat, Hervé Dupont

**Affiliations:** 1 Neurosurgical Intensive Care Unit, Department of Anaesthesiology and Critical Care Medicine, Amiens University Hospital, Place Victor Pauchet, 80054, Amiens, France; 2 Department of Neurosurgery, Amiens University Hospital, Place Victor Pauchet, 80054, Amiens, France; 3 INSERM U-1088, Jules Verne University of Picardy, 3 rue des Louvels, 80036, Amiens, France

## Abstract

**Introduction:**

The aim of this study was to evaluate the prognostic value of optic nerve sheath diameter (ONSD) measured on the initial brain computed tomography (CT) scan for intensive care unit (ICU) mortality in severe traumatic brain injury (TBI) patients.

**Methods:**

A prospective observational study of all severe TBI patients admitted to a neurosurgical ICU (over a 10-month period). Demographic and clinical data and brain CT scan results were recorded. ONSD for each eye was measured on the initial CT scan. The group of ICU survivors was compared to non-survivors. Glasgow Outcome Scale (GOS) was evaluated six months after ICU discharge.

**Results:**

Seventy-seven patients were included (age: 43 ± 18; 81% males; mean Injury Severity Score: 35 ± 15; ICU mortality: 28.5% (*n *= 22)). Mean ONSD on the initial brain CT scan was 7.8 ± 0.1 mm in non-survivors vs. 6.8 ± 0.1 mm in survivors (*P *< 0.001). The operative value of ONSD was a good predictor of mortality (area under the curve: 0.805). An ONSD cutoff ≥ 7.3 had a sensitivity of 86.4% and a specificity of 74.6% and was independently associated with mortality in this population (adjusted odds ratio 95% confidence interval: 22.7 (3.2 to 159.6), *P *= 0.002). There was a relationship between initial ONSD values and six-month GOS (*P *= 0.03).

**Conclusions:**

ONSD measured on the initial brain CT scan is independently associated with ICU mortality rate (when ≥ 7.3 mm) in severe TBI patients.

## Introduction

Severe traumatic brain injury (TBI) is a leading cause of death and disability [1,2]. A review of 23 surveys carried out in Europe estimated the annual incidence of TBI to be 235 per 100,000, with a mortality rate of about 15.4 per 100,000 - mainly due to motor vehicle accidents or falls [2]. In another recent survey, mortality in the United States was reportedly higher (18.4 per 100,000) [1]. Post-TBI disability is a major concern and its prevalence is probably underestimated. It is important to optimize the pre-admission care of TBI patients [3]. Improving a patient's overall prognosis requires mobilization of healthcare system human and material resources for the most seriously injured patients. Many factors have been reported to influence the prognosis of TBI, including age, gender, severity of injury, co-morbidities, concomitant use of anticoagulants, secondary insults, the initial Glasgow Coma Scale (GCS) score, the motor score, pupil reactivity, the type of lesion visualized on brain computed tomography (CT) scan, changes in intracranial pressure (ICP) and blood levels of specific proteins. Two prognostic scores for TBI patients on admission have been developed on the basis of the large, prospective IMPACT and CRASH databases [4,5]. However, these scores are complex, require numerous data and are not easy to perform in the emergency unit. In this context, there is a need for a clearly relevant parameter with prognostic value for patients in the initial phase of TBI. The optic nerve sheath is the most accessible part of the brain meninges [6]. A strong relationship has been reported between optic nerve sheath diameter (ONSD, as evaluated by ultrasound) and ICP [7-10]. Severe TBI patients admitted to the ICU generally undergo at least one initial brain CT scan. However, to the best of our knowledge, no study has focused on the initial ONSD measurement. The aim of the present study was therefore to evaluate the relationship between ONSD on the initial brain CT scan and the outcome of severe TBI patients admitted to the ICU.

## Materials and methods

### Study design

This prospective, single-centre, observational study was conducted in a neurosurgical ICU over a 10-month period (October 2009 to July 2010). The inclusion criterion was severe TBI in patients over the age of 18 admitted to the ICU. The main exclusion criteria were: lack of initial brain CT scan or millimetre-scale images or sequences, facial trauma affecting the orbits and/or eyeballs, pre-existing ocular disease affecting the optic nerve and/or orbital cavity and hyperthyroidism with exophthalmia. The primary objective of the study was to assess the potential relationship between mean ONSD measured on the initial brain CT scan and ICU mortality.

An independent ethics committee for non-interventional research of the Amiens University Hospital approved the study protocol. In view of the study's non-interventional, observational nature, the committee did not require participants to provide their informed consent.

### Traumatic brain injury management

The ICU management protocol for TBI patients is based on the Brain Trauma Foundation guidelines [11]. Standard criteria were used for surgical evacuation and mannitol use before surgery [12]. Hypertonic saline, decompressive craniectomy and hypothermia are not used in our institution. ICP monitoring is performed only in the more severe patients with subnormal CT. The target cerebral perfusion pressure was that recommended in French guidelines (> 70 mmHg) [13].

### Data collection

Data concerning age, gender, Injury Severity Score (ISS) [14], GCS score on admission and clinical neurological examination were collected. The presence of subarachnoid or intraventricular haemorrhage, basal cistern compression, cortical sulcus effacement or midline shift of more than 5 mm on the initial CT scan was noted. The Marshall score was then calculated, as previously described [15]. The frequencies of emergency neurosurgery and ICP monitoring were noted. The following clinical biochemical parameters were collected on admission to the ICU: serum sodium, glucose and haemoglobin, PaO_2_/FiO_2 _and PaCO_2_. Mortality in the ICU was noted. For survivors, the Glasgow Outcome Scale (GOS) was assessed six months after discharge [16]. The length of stay in the ICU was also recorded.

### Optic nerve sheath diameter measurement

**Figure 1:**
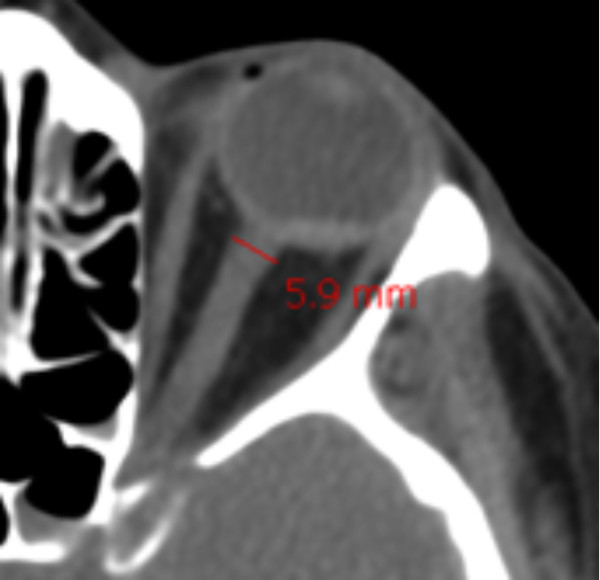
**Optic nerve sheath diameter measurement on the initial brain computed tomography scan (using DxMM™ software)**.

The same physician (AL) examined all initial CT scans included in the study. He was blinded to the patient's medical history, the circumstances of the TBI and the patient's severity scores at the time of measurement. All CTs were performed before surgery, when needed. ONSD was measured on the first brain CT scan recorded prior to the patient's admission to the ICU. Brain CT scan was performed with a series of millimetre slices (one slice every 0.6 mm). As for ultrasound or magnetic resonance imaging (MRI), ONSD was measured at a distance of 3 mm behind the eyeball, immediately below the sclera [17,18] using DxMM software (DICOM v6.5 SP5, Medasys™, Gif-sur-Yvette, France). ONSD was measured from one side of the optic nerve sheath to the other (as shown in Figure 1) as a section through the centre of the optic nerve. The diameters measured for the patient's left and right eyes were averaged to yield the mean value. Before starting the study, intra- and inter-observer variabilities were evaluated on three different physicians and were calculated retrospectively by analysis of brain CT scans. Intra-observer variability of ONSD measurement was 2% ± 2% (Cronbach's α coefficient = 0.996) and inter-observer variability was 6% ± 5% (Cronbach's α coefficient = 0.893).

### Statistical analysis

Categorical parameters are expressed as number and frequency. Quantitative parameters are expressed as mean ± standard deviation (when normally distributed) or median with interquartile range. ICU survivors were compared to non-survivors. Categorical parameters were compared by chi-square test (using Yates' correction) or, when necessary, Fisher's exact test. Quantitative parameters were compared with a two-tailed *t *test (when normally distributed) or with a Mann-Whitney *U *test. Receiver operating characteristic (ROC) curves were plotted to determine the performance of ONSD to predict mortality. Once the best cutoff value had been determined graphically, the sensitivity, specificity, positive and negative predictive values and positive and negative likelihood ratios were calculated with their 95% confidence intervals (CIs). A multivariate, stepwise, logistic regression model (backward Wald models) was then constructed to identify any independent factors for ICU mortality in severe TBI patients. Adjusted odds ratios (AORs) and their 95% CIs were calculated. The model's entry and exit values were set at 0.05 and 0.1, respectively. The constant (intercept) was only included in the model when it was statistically significant [19]. The Hosmer-Lemeshow test was used to assess the model's goodness of fit [19]. The statistical significance of individual regression coefficients was tested using the Wald chi-square test [19]. The model's predicted probabilities were validated with the *c *statistic (corresponding to the area under the model's curve) [19]. A Kruskal-Wallis test was used to compare initial ONSD and GOS values six months after ICU discharge for survivors. The limit for statistical significance was *P *≤ 0.05. Statistical analysis was performed with PASW Statistics 18 software (IBM, Chicago, IL, USA).

## Results

Eighty-five adult TBI patients were admitted to the Amiens University Hospital ICU (Amiens, France) between October 2009 and July 2010. Four patients under the age of 18 were not included. Three individuals with traumatic orbital haematoma and one patient lacking an initial brain CT were excluded. A total of 77 patients were therefore included in the study (mean age: 43 ± 18; 81% males). TBI was mainly due to falls (47%) and motor vehicle accidents (43%). The time interval between trauma and the first brain CT was always < 3 hours. One-third of patients had a blood alcohol concentration over the legal limit in France. The mean ISS was 35 ± 15. One-third of patients had been initially treated in a primary care hospital. The initial GCS score was 7.2 ± 4.2. Twenty-four patients (31%) underwent neurosurgical evacuation of intracranial haematoma prior to admission to the neurosurgical ICU. Norepinephrine was administered in 13 patients (17%). The initial CT scan revealed traumatic, subarachnoid haemorrhage in 38 patients (49%), intraventricular haemorrhage in 13 patients (17%) and a midline shift in 30 patients (39%) and yielded a mean Marshall score of 3.8 ± 2.1. Invasive ICP monitoring was initiated in seven patients (9%). The mean length of stay in the ICU was 12 ± 14 days.

A strong relationship between CT signs suggestive of elevated ICP and ONSD values was observed: basal cisternal effacement: 7.5 ± 1.1 mm vs. 6.9 ± 0.9 (*P *= 0.02); midline shift ≥ 5 mm: 7.5 mm ± 0.9 vs. 6.8 ± 1.1 (*P *= 0.0002) and diffuse sulcal effacement: 7.4 mm ± 1.1 vs. 6.6 ± 0.9 (*P *= 0.001). A significant positive correlation was observed between ONSD and Marshall score (*P *= 0.01). ONSD was 7.6 mm ± 1.0 vs. 6.9 ± 1.0 (*P *= 0.01) in case of non-reactive pupils, 7.3 mm ± 1.1 vs. 6.9 ± 1.0 (*P *= 0.26) in the case of anisocoria and 7.6 mm ± 1.0 vs. 6.9 ± 1.1 (*P *= 0.03) in patients requiring surgical evacuation.

**Figure 2:**
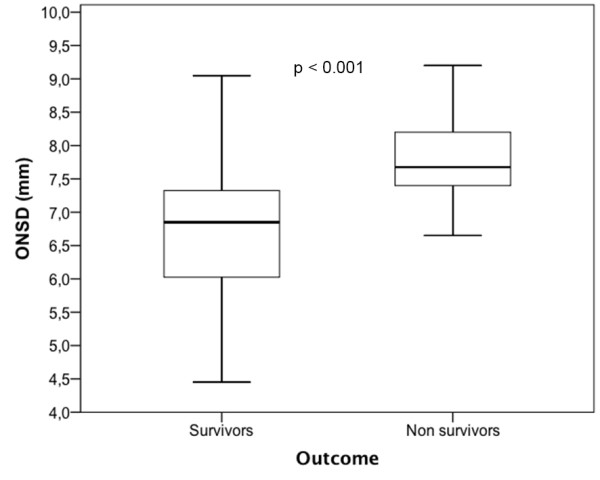
**Box plot of optic nerve sheath diameter (mm) comparing survivors and non-survivors among severe traumatic brain injury patients**.

**Figure 3:**
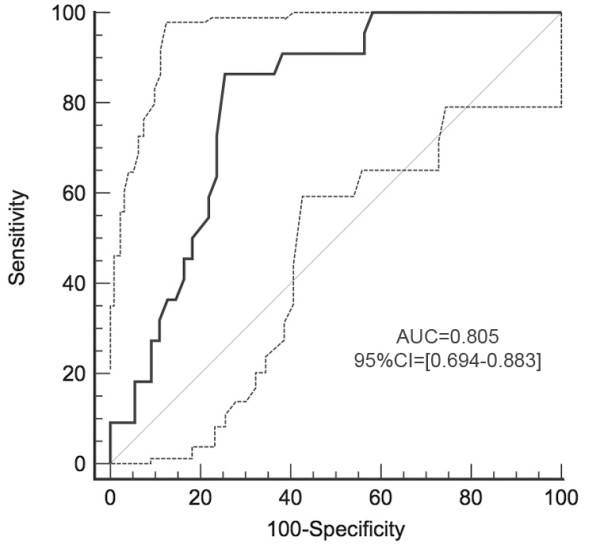
**Receiver-operator characteristic curves of the performance of optical nerve sheath diameter to predict mortality**. Dotted lines represent 95% confidence intervals (95% CI) of each point. AUC, area under the curve.

The ICU mortality rate was 28.6% (22 patients). Among the survivors, 46 were extubated (82%) and nine were tracheotomised (16%) during their stay in the ICU. The cause of death was brain death in 16 patients (72.7%), multiple organ failure in four (18.2%) and miscellaneous for two (9.1%). Demographic variables, TBI severity, clinical signs and outcomes are presented in Table 1. Briefly, non-survivors were older (*P *= 0.005), with a higher ISS (*P *= 0.02), a lower initial Glasgow Coma Scale (*P *= 0.03) and more frequent pupil reactivity abnormalities (*P *< 0.001). Initial brain CT scan results and clinical biochemical parameters are presented in Table 2. Compared with survivors, non-survivors presented more signs of intracranial hypertension, a higher incidence of subarachnoid haemorrhage (*P *= 0.04) and higher ONSD (6.8 ± 0.1 mm vs. 7.8 ± 0.1 mm, *P *< 0.001), as shown in Figure 2. A ROC curve was plotted to assess the prognostic value of ONSD (Figure 3). The area under the curve was 0.805 with a 95% CI of 0.694 to 0.883. A cutoff ≥ 7.3 mm had a sensitivity of 86.4% (65.1% to 97.1%), a specificity of 74.6% (61.0% to 85.3%), a positive predictive value of 57.6% (38.9% to 74.8%), a negative predictive value of 93.2% (81.3% to 98.6%), a positive likelihood ratio of 3.4 (2.7 to 4.3) and a negative likelihood ratio of 0.2 (0.1 to 0.6). Four parameters were independently associated with mortality: age > 32 years (AOR: 18.1, 95% CI: (1.6 to 201.2), *P *= 0.02), anisocoria on admission (AOR: 10.4; 95% CI: (1.6 to 65.1), *P *= 0.01), basal cistern compression on CT scan (AOR: 10; 95% CI: (1.9 to 53.4), *P *= 0.005) and ONSD ≥ 7.3 mm (AOR: 22.7; 95% CI: (3.2 to 159.6), *P *= 0.002). The Wald chi-square value for the model was 52.7 (df = 4, *P *= 0.0001). The goodness of fit of the model was not significant (Hosmer-Lemeshow chi-square = 3.9, ddl = 6, *P *= 0.69). The *c*-statistic of the model was 0.96 with 95% CI (0.92 to 0.99).

**Table 1:** The main clinical signs on admission in survivors and non-survivors.

	Survivors	Non-survivors	*P *value
	(*n *= 55)	(*n *= 22)	
Age (year)	39 ± 17	52 ± 16	0.005
Male gender	43 (78.2%)	19 (80.4%)	0.41
ISS	29 [16]	32 [32]	0.02
Initial GCS score	7 [7]	5 [6]	0.03
Pupil reactivity	44 (80%)	12 (55%)	0.02
Anisocoria	13 (24%)	15 (68%)	< 0.001
Suspected EICP	11 (20%)	8 (36%)	0.13
ICU admission			
MAP (mmHg)	90 [18]	89 [36]	0.8
HR (bpm)	90 ± 3	79 ± 6	0.09
Temperature (°C)	37 [2]	36 [2]	0.33
Neurosurgical operation	17 (31%)	7 (32%)	0.93
Length of stay in the ICU (days)	15 ± 15	5 ± 3	0.002

Results are presented as number (proportion), mean ± standard deviation or median [interquartile range] according to the normality of distribution. ISS, Injury Severity Score; GCS, Glasgow Coma Scale; EICP, elevated intracranial pressure; MAP, mean arterial pressure; HR, heart rate.

**Table 2:** The main brain CT scan and clinical biochemical parameters in survivors and non-survivors.

	Survivors	Non-survivors	*P *value
	(*n *= 55)	(*n *= 22)	
Initial CT scan			
Basal cistern compression	7 (13%)	15 (68%)	< 0.001
Midline shift ≥ 5 mm	17 (31%)	13 (59%)	0.04
Cortical sulcus effacement	26 (47%)	20 (91%)	< 0.001
Intraventricular haemorrhage	8 (15%)	5 (22%)	0.5
Subarachnoid haemorrhage	23 (42%)	15 (68%)	0.04
Marshall score	3 [5]	5 [3]	0.22
Optic nerve sheath diameter (mm)	6.8 ± 0.1	7.8 ± 0.1	< 0.001
Clinical biochemical parameters			
Serum sodium (mmol.l^-1^)	139 ± 4	138 ± 5	0.51
Glycaemia (mmol.l^-1^)	7.3 [2.5]	8.9 [4.6]	0.008
PaO_2_/FiO_2_	332 [126]	174 [118]	0.13
PaCO_2 _(mmHg)	38.4 [9.9]	40.5 [10.5]	0.69
Haemoglobin (g.dl^-1^)	13.0 [2.8]	12.8 [3.3]	0.68

Results are presented as number (proportion), mean ± standard deviation or median [interquartile range] according to the normality of distribution. CT, computed tomography.

**Figure 4:**
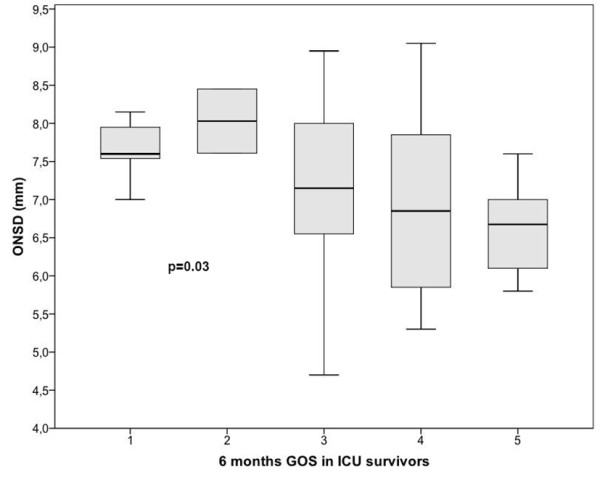
**Box plot of optic nerve sheath diameter (mm) according to the Glasgow Outcome Scale (GOS) determined six months after discharge from ICU for 55 survivors (*P *= 0.03)**.

The GOS six months after discharge for the 55 ICU survivors was as follows: GOS 1 (*n *= 5, 9.1%), GOS 2 (*n *= 2, 3.6%), GOS 3 (17, 30.1%), GOS 4 (*n *= 13, 23.6%) and GOS 5 (*n *= 18, 32.7%). Overall six-month mortality in severe TBI was 35%. A statistically significant relationship was observed between GOS and initial ONSD measurement, as shown in Figure 4 (*P *= 0.03).

## Discussion

This study demonstrated a strong, independent relationship between ONSD on the initial brain CT scan and the mortality rate among severe TBI patients admitted to a neurosurgical ICU.

Mean ONSD in this study was much higher than that previously reported under various measuring conditions, even in patients with severe TBI. However, these results are in accordance with previous studies measuring ONSD on CT. The first study measured ONSD in 100 healthy volunteers [20] and found a mean ONSD of 4.4 mm with a 95% CI of 3.2 to 5.6. These values in healthy volunteers are higher than those proposed for the detection of elevated ICP by sonography. More recently, ONSD was evaluated on CT in normal-tension glaucoma patients compared to that of healthy volunteers [21] and revealed a mean ONSD of 8.0 ± 1.0 in normal-tension glaucoma patients and 6.2 ± 0.9 in healthy volunteers. Experimentally, in an optic nerve model obtained from cadaveric donors without neurological injury, a clear relationship was demonstrated between increased intracranial pressure and increased ONSD, but with ONSD values at 6.7 mm with higher pressures [22]. However, this study was purely experimental and was not conducted on injured patients under pathophysiological conditions. ONSD measurements have been more frequently reported with ultrasound. ONSD values greater than 7.0 mm have been reported in TBI patients with intracranial hypertension [23]. However, in a retrospective study, ONSD ≥ 5.0 mm was proposed to detect elevated ICP [24]. The inter-observer and intra-observer variations of ultrasound measurement of ONSD were recently calculated to be 0.5 mm and 0.2 mm, respectively [25]. These results are very similar to those reported in the present study based on CT measurement of ONSD. MRI has also been proposed to measure ONSD with a lower range of values than those reported in this study [18]. However, the limited accuracy of MRI with thicker brain slices may underestimate the real value of ONSD. With MRI measurement, errors have also been reported to be 1.3 to 1.5 mm [26]. One MRI study on ONSD in TBI patients with invasive ICP has been published [18]. The ONSD was significantly greater in TBI patients with raised ICP (> 20 mmHg; 6.31 +/- 0.50 mm) than in those with ICP of 20 mmHg or less (5.29 +/- 0.48 mm) or in healthy volunteers (5.08 +/- 0.52 mm). A study comparing MRI and B sonography in healthy subjects [27] showed that sonography underestimated ONSD: 5.72 mm (5.51 to 5.93) for MRI vs. 4.08 (3.4 to 4.3) for sonography. A recent evaluation of ONSD measured by MRI in astronauts exposed to microgravity reported ONSD values of 6.5 ± 1.0 mm [28]. The ONSD values measured by CT and MRI are higher than those reported with sonography. The ONSD measurements reported in the present study appear to be accurate in view of the low intra- and inter-observer variability determined prior to the study. A good correlation has been reported between CT scan and ultrasonography in healthy subjects [29]. However, the previous study overestimated ONSD by more than 10% with CT compared to sonography [29]. The mean ONSD in healthy subjects has been reported to be 5.5 mm [30], very close to the limit for elevated ICP reported for sonography [31], emphasising the differences in the measurements between these two methods.

Experimentally, Hansen *et al*. showed a correlation between ONSD and ICP and ICP variations [6,9]. Other authors have used bedside transorbital sonography to assess ONSD in hydrocephalic patients [32]. Many researchers have investigated the significance of this parameter as a means of detecting intracranial hypertension [10,17] in patients with TBI [8,33], thereby allowing rapid triage in the emergency unit [34]. In the emergency department, an US evaluation of ONSD ≥ 5.0 mm was associated with CT findings of elevated ICP ([35]. A link between MRI-determined ONSD and elevated ICP has also been reported [18,36]. However, to the best of our knowledge, no study has yet focused on brain CT scan that is routinely performed in severe TBI patients. ONSD is much easier to measure on CT scan than with sonography due to the good reproducibility of CT and the lack of a learning curve. Furthermore, with the development of telemedicine, tertiary reference centres now have access to the initial CT scan [37]. ONSD measurement may facilitate transfer decisions. The relationship between intracranial hypertension and prognosis has been widely demonstrated [38-41]. Furthermore, ICP monitoring *per se *has never been found to be associated with improved outcomes in severe TBI patients [42] compared to clinical and radiological evaluation. When combined with other parameters, the initial measurement of ONSD may be clinically useful in the early diagnosis setting and to rapidly initiate aggressive treatment of suspected elevated ICP.

Interpretation of our data is subject to several limitations. First, the sample size may be considered to be small. We nevertheless included all consecutive patients over the study period. Some patients with less severe forms of TBI may have been hospitalized in primary care hospital ICUs without referral to our university hospital after CT imaging. In contrast, high-severity TBI patients rapidly progressing to brain death would not have been considered for transfer due to local multi-organ donation procedures. Although this aspect clearly constitutes a limitation, the measurement technique is very easy to implement and depends more on the software tools than the physician's ability. Furthermore, all brain CT scans are now performed in millimetre slices, enabling easy measurement of ONSD. The inter- and intra-observer variability of ONSD measurement was in accordance with the results reported in other studies, with very good reliability. Finally, the rate of ICP monitoring was very low in these severe TBI patients. This is another limitation of the study despite recent studies showing similar outcomes with ICP management versus clinical and radiological evaluation [42]. Brain CT scan ONSD measurements cannot be considered to be a monitoring tool for severe TBI patients. This study was not designed to evaluate this point, but only to consider available data before ICU admission. ONSD monitoring is much easier and less invasive by bedside sonography.

## Conclusions

This study demonstrated a relationship between ONSD measured on the initial brain CT scan of severe TBI patients and ICU mortality rate. This simple, CT-based measurement could be of value in initial triage of severe TBI patients when transferring images from primary hospitals to reference centres. However, these results need to be validated in a larger cohort of patients.

## Key messages

• There is a relationship between ONSD measured on admission brain CT scan in severe TBI patients and outcome. However, high ONSD does not exclude good outcome.

• CT scan is readily available in all emergency departments and ONSD measurement may facilitate the decision to transfer the patient to referral centres. ONSD can be easily measured with the digital tools available on most CT consoles. This estimation should be confirmed with normative data like ultrasound.

• ONSD could be integrated into the initial management of severe TBI patients together with other clinical, radiological and laboratory parameters, especially after ONSD validation by other methods.

• These results need to be validated in a larger cohort of patients.

## Abbreviations

AOR: adjusted odds ratio; CI: confidence interval; CT: computed tomography; GCS: Glasgow Coma Scale; GOS: Glasgow Outcome Scale; ICP: intracranial pressure; ISS: Injury Severity Score; MRI: magnetic resonance imaging; ONSD: optical nerve sheath diameter; ROC: receiver operating characteristic; TBI: traumatic brain injury.

## Competing interests

The authors declare that they have no competing interests with this study.

## Authors' contributions

AL, PJ and HD designed the study. AL, FD and BL were responsible for enrolment and data collection. AL, PJ, BL, HD and JP performed data analysis. HD was responsible for statistical analysis and revision of the manuscript. All authors were involved in writing of the manuscript and approved the final version.
